# Calibration, Conversion, and Quantitative Multi-Layer Inversion of Multi-Coil Rigid-Boom Electromagnetic Induction Data

**DOI:** 10.3390/s19214753

**Published:** 2019-11-01

**Authors:** Christian von Hebel, Jan van der Kruk, Johan A. Huisman, Achim Mester, Daniel Altdorff, Anthony L. Endres, Egon Zimmermann, Sarah Garré, Harry Vereecken

**Affiliations:** 1 Institute of Bio- and Geoscience, Agrosphere (IBG-3), Forschungszentrum Jülich GmbH, 52428 Jülich, Germany; s.huisman@fz-juelich.de (J.A.H.);; 2 Centre for High-Performance Scientific Computing in Terrestrial Systems (TerrSys), Forschungszentrum Jülich GmbH, 52428 Jülich, Germany; 3 Central Institute for Engineering, Elektronics and Analytics, Electronic Systems (ZEA-2), Forschungszentrum Jülich GmbH, 52428 Jülich, Germany; a.mester@fz-juelich.de (A.M.); e.zimmermann@fz-juelich.de (E.Z.); 4 Boreal Ecosystem Research Initiative, Memorial University, Corner Brook, NL A2H 5G4, Canada; daltdorff@grenfell.mun.ca; 5 Earth and Environmental Science, University of Waterloo, Waterloo, ON N2L 3G1, Canada; alendres@uwaterloo.ca; 6 Gembloux Agro-Bio Tech, Liège University, 5030 Gembloux, Belgium; sarah.garre@uliege.be

**Keywords:** electromagnetic induction (EMI), EMI data calibration, accurate magnetic field data conversion, quantitative layered electrical conductivity inversion, toward linking depth-specific large-scale quasi-3D inversion results with geo-observational data

## Abstract

Multi-coil electromagnetic induction (EMI) systems induce magnetic fields below and above the subsurface. The resulting magnetic field is measured at multiple coils increasingly separated from the transmitter in a rigid boom. This field relates to the subsurface apparent electrical conductivity (σ_a_), and σ_a_ represents an average value for the depth range investigated with a specific coil separation and orientation. Multi-coil EMI data can be inverted to obtain layered bulk electrical conductivity models. However, above-ground stationary influences alter the signal and the inversion results can be unreliable. This study proposes an improved data processing chain, including EMI data calibration, conversion, and inversion. For the calibration of σ_a_, three direct current resistivity techniques are compared: Electrical resistivity tomography with Dipole-Dipole and Schlumberger electrode arrays and vertical electrical soundings. All three methods obtained robust calibration results. The Dipole-Dipole-based calibration proved stable upon testing on different soil types. To further improve accuracy, we propose a non-linear exact EMI conversion to convert the magnetic field to σ_a_. The complete processing workflow provides accurate and quantitative EMI data and the inversions reliable estimates of the intrinsic electrical conductivities. This improves the ability to combine EMI with, e.g., remote sensing, and the use of EMI for monitoring purposes.

## 1. Introduction

The critical zone contains water resources, influences the climate, supports ecosystems, and sustains anthropogenic activities such as agriculture, infrastructure, and waste disposal. An improved understanding of the critical zone is essential, and detailed knowledge of the subsurface structural patterns is of utmost importance for critical zone investigations and modeling. Geophysical tools can provide such information, and therefore improve our understanding of processes occurring in that critical zone. The upper few meters of the subsurface can be investigated using minimally-invasive or non-invasive geoelectrical methods, such as ground penetrating radar, electromagnetic induction (EMI), and direct current (DC) methods. Both EMI and the DC methods are sensitive to the electrical conductivity of the subsurface, which depends on subsurface properties such as clay and mineral content, soil texture, water content, and salinity. The DC resistivity method injects currents into the subsurface using grounded electrodes inserted into the soil surface. The EMI technique contactlessly generates the currents in the subsurface by making use of induction phenomena. Especially for large-scale measurements, portable and mobile multi-coil EMI systems are often used. Such systems provide apparent electrical conductivity (σ_a_) values that can be used to infer the soil water content distribution within a catchment [1,2], and/or upscale other soil properties [3,4] up to the sub-continental scale [5]. In precision agriculture, σ_a_ patterns help to optimize fertilizer and irrigation application [6,7], to investigate long-term treatment effects [8], to investigate plant and soil interactions [9,10,11,12], and to test the ability of plants to grow in saline soil conditions [13]. The spatial distribution of σ_a_ can also be used to identify buried cables [14], flood embankments [15], and features within morphological, geological, and geoarchaelogical units [16,17,18]. Recently, Heil et al. [19] provided a comprehensive overview of σ_a_ mapping applications focusing on the two-coil EM38 (Geonics Ltd., Mississauga, Canada) system.

Multi-coil rigid-boom EMI systems use one transmitter (Tx) and up to six receiver coils (Rx). Examples are the CMD-MiniExplorer (GF-Instruments, Brno, Czech Republic) or the DualEM-421s (DualEM Ltd., Milton, ON, Canada). The coils are rigidly connected in one boom with fixed separations [20,21] ranging between 0.3 and 4 m. The increasing coils simultaneously measure σ_a_ over multiple overlapping increasing depth ranges, which enables inversion using the combined data set to obtain one-dimensional (1D) layered electrical conductivity models along transects [22,23,24]. At a larger scale, von Hebel et al. [25] presented a quasi-3D (three-dimensional) EMI data inversion approach, which was subsequently used to investigate soil structures associated with buried paleo-river channels that provide vital plant resources at specific soil depths [12]. Other studies used multi-coil EMI data inversion to explore the structure and geometry of paleo-river channels [26,27] or inverted multi-frequency EMI data to study contaminant distributions [28] and Artic sea-ice structures [29]. However, inversion approaches using EMI data are relatively new compared to the inversion of DC data. 

The DC resistivity method was first used in geophysics by the Schlumberger brothers in 1912. They used four electrodes; two (source electrodes) to inject current into the ground and two (receiver electrodes) to meaure the potential. By increasing the distance bewteen source and receiver around a midpoint, the depth of investigation increases. This is called vertical electrical sounding (VES). If one combines measurements of multiple electrodes in various combinations, many depth ranges and horizontal variations of σ_a_ can be measured along a transect. This is called electrical resistivity tomography (ERT). In order to obtain the intrinsic electrical conductivities of certain layers, an inversion has to be performed. DC resistivity inversion is well established [30] and, up till now, more reliable than EMI inversion results based on a comparison with ground-truth data [31,32]. The imaging capabilities of multi-coil EMI systems therefore needs further improvements.

A major drawback of many EMI surveys is that the measured σ_a_ values are affected by systematic measurement errors and can thus only be used in a qualitative way [33]. Besides quickly changing influences such as solar flares, humidity, and temperature changes during the survey that may distort EMI measurements [34,35], differences in σ_a_ already occur when different operators measure along the same transect [36]. In addition, some EMI systems require a regular zero-leveling during the survey, such that erroneous σ_a_ readings may occur due to user and onsite calibration bias [36,37,38]. The need for regular zero-leveling has been removed in the more recent multi-coil EMI systems. These systems now provide unbiased and stable, but nevertheless qualitative, σ_a_ values due to stationary influences associated with the proximity of electric cables, data loggers, and global positioning system (GPS) device close to the EMI system. These stationary influences need to be investigated and corrected through calibration to obtain quantitative σ_a_ values suitable for inverting the intrinsic electrical conductivity distribution.

To calibrate σ_a_, the EMI system can be lifted at multiple elevations to simultaneously invert for a subsurface model and the calibration factors [39]. Also, a linear regression between independently obtained σ_a_ values and those measured with the EMI system can be used by taking pointwise information of σ depth profiles along extracted soil cores [40] or by predicitng σ_a_ values based on direct current ERT data [41]. This ERT-based calibration approach has successfully corrected σ_a_ values measured with EMI and resulted in reliable inversion results in a range of studies [12,24,33,41,42,43,44,45]. Nevertheless, the calibration of σ_a_ measured with EMI using direct current methods can be seen critically. Both EMI and DC measurements rely on different electrical couplings and use different frequencies (see also Appendix A). In addition, DC inversions are inherently non-unique and can thus bias the calibration results. On the other hand, DC methods and the EMI technique are sensitive to subsurface electrical conductivity [46], which typically shows a neglible dependency on the frequency in the Hz to kHz range, where DC methods and EMI operate (see, e.g., Lower et al. [47]). In addition, the sensitivity curves for DC methods and horizontal coplanar EMI coils are similar for a horizontally layered subsurface. They show a maximum at a certain depth, as well as zero sensitivity at the surface and larger depths [48,49]. Therefore, the information content of a series of DC measurements should be similar to that of multi-coil EMI data. Nevertheless, there are still open questions about the use of DC methods for EMI calibration. In particular, the most appropriate electrode array for ERT data acquisition, the general stability of the calibration approach, and the possible use of VES data measured at few locations instead of an ERT line as proposed by von Hebel et al. [12] and Thiesson et al. [50] still need to be investigated.

The σ_a_ values provided by rigid-boom EMI systems are a user-friendly representation of the complex measurements. EMI systems transmit a primary magnetic field through the Tx and receive the superposition of the primary and a secondary magnetic field generated by electrically conductive material. Mathematically, the received resultant magnetic field contains an imaginary (quadrature, Q) and a real (in-phase) component. The Q component can be converted to σ_a_. For this, often a so-called low induction number (LIN) approximation [48] is used. The multi-coil rigid-boom EMI systems are designed to work under low induction numbers by using relatively small (up to 4 m) separated coils and low frequencies [48]. However, the LIN-approximation is valid for a homogeneous subsurface with low electrical conductivity (<12 mS/m [51,52]). To overcome this limitation, several methods have been introduced to relate Q and σ_a_ for rigid-boom EMI systems (e.g., [51,53,54]), for multi-frequency EMI systems (e.g., [55]), and for large-loop EMI systems (e.g., [56,57]). However, it is still often assumed that rigid-boom EMI systems use the LIN-approximation to obtain σ_a_ inside the system, which is not generally true. For example, the CMD-MiniExplorer bases the system design on low induction numbers (small s, low frequency), but does not use the LIN-approximation for arithmetical calculation [58], and the GEM-2 (Geophex Ltd., Raleigh, NC, USA) obtains σ_a_ using a full physical model. Thus, a user may choose/find system specific solutions to improve the conversion accuracy.

**Figure 1:**
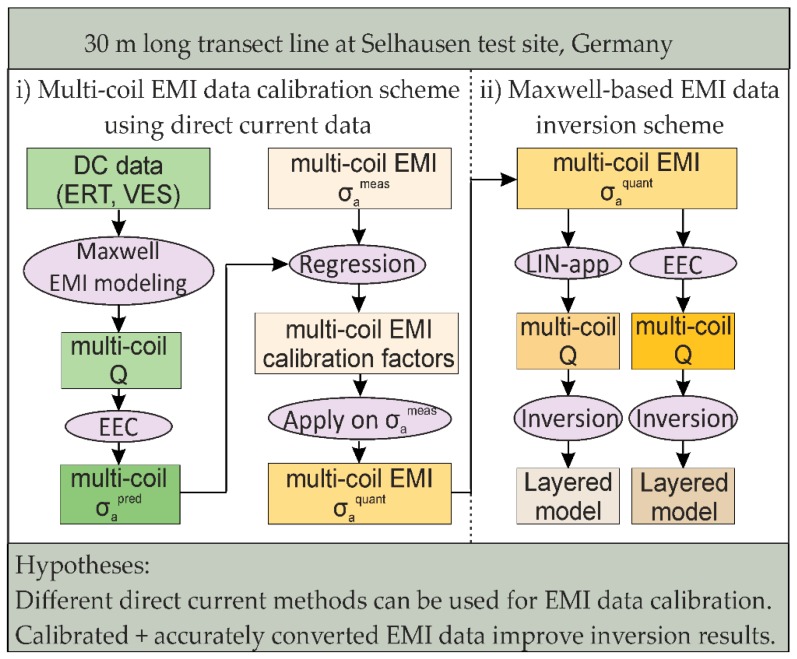
Flowchart of EMI data calibration and conversion strategies used for inversion. The calibration is based on direct current data, which are used to predict σ_a_. A linear regression between measured and predicted σ_a_ provides multiplicative and additive calibration factors that are used to obtain quantitative σ_a_. These are converted to the quadrature (Q) component of the magnetic field ratios using the low induction number approximation (LIN app) and the exact EMI conversion (EEC). The respective Q values are then inverted to obtain a layered electrical conductivity model of the subsurface. Abbreviations: EMI, electromagnetic induction; DC, direct current; ERT, electrical resistivity tomography; VES, vertical electrical sounding.

Summarizing, Figure 1 hypothesizes that (1) different direct current methods can be equally used to calibrate the σ_a_ values measured with an EMI system, and that (2) calibrated and accurately converted EMI data obtain reliable inversion results. In particular, we aim to establish a novel processing chain for multi-coil rigid-boom EMI system data to enable accurate and quantitative subsurface characterization. Starting with calibration, EMI and DC data are measured, where the latter are used to predict σ_a_. A linear regression with the σ_a_ measured with EMI obtains the calibration factors that are used to obtain quantitative σ_a_ values. To then obtain a layered subsurface model using the Maxwell-based EMI data inversion scheme, the quantitative σ_a_ values are converted to Q using the LIN-approximation, and the exact EMI conversion (EEC) that will be introduced here. 

The overall objective of this research paper is to analyze the effect of different calibration procedures using DC methods, as well as the conversion strategies on subsequent EMI data inversions. In the next section, the EMI forward modeling and data inversion procedure is described. Also, the non-linear exact EMI conversion approach is presented and compared to the LIN-approximation. Then, the calibration of σ_a_ measured with EMI using direct current data is explained. In the following results and discussion section, we investigate the influences that alter EMI measurements, and study the use of different DC methods for EMI calibration. We then evaluate the stability of the calibration for a broad range of soils using data acquired at eighteen different sites. Finally, we analyze the effect of the calibration and conversion strategies within layered EMI data inversions. 

## 2. Electromagnetic Induction Data Modeling, Conversion, Calibration, and Inversion

### 2.1. EMI Principle

**Figure 2:**
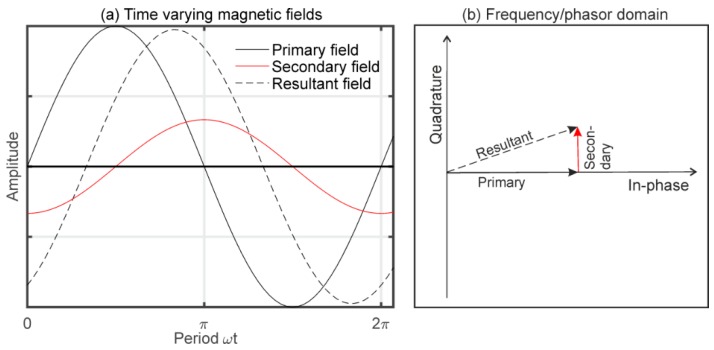
(**a**) The time varying primary magnetic field induces a secondary field that superimposes to a resultant magnetic field. (**b**) This oscillating field can be represented in the frequency domain, where it draws a phasor line that is composed of an in-phase and a quadrature component.

A rigid-boom EMI system sends an alternating current at one frequency (~kHz) through the transmitter (Tx) coil to generate a primary magnetic field, as displayed in Figure 2a. This induces eddy currents in an electrically conductive subsurface, which in turn generate a secondary magnetic field. This is superimposed with the primary field and a resulting magnetic field is measured at the receiver coils (Rx). In the frequency domain, as shown in Figure 2b, the resultant field draws a phasor containing an in-phase and a quadrature (Q) component. 

The in-phase component contains information on the magnetic susceptibility and, to a lesser degree, on the electrical conductivity. EMI systems with short (≤4 m) Tx–Rx separations are generally incapable of measuring the in-phase with sufficient accuracy [59]. The Q component that dominates the signal for low induction numbers is related to the subsurface electrical conductivity and can be measured with acceptable accuracy [60,61]. 

#### 2.1.1. Maxwell-Based EMI Forward Models for Homogeneous and Layered Subsurfaces

The dimensionless induction number (N_b_) is defined as Nb= sδ, where s is the coil separation and δ is the skin depth given by:

(1) $$\delta =\;\sqrt{\frac{2}{\omega \mu_{0}\sigma }}$$

Here, σ is the electrical conductivity, µ_0_ is the magnetic permeability of free-space, ω = 2πf is the angular frequency, and f is the frequency of the primary magnetic field. The skin depth essentially determines the depth of current flow. At δ, the magnetic field strength is 1/*e* (*e* ≈ 2.7183) or 37% of the initial value. The quasi-static electromagnetic fields generated at low EMI frequency are described by the propagation constant $\gamma =\sqrt{j\omega \mu_{0}\sigma }$. This depends on σ, since the magnetic constant (µ_0_) and the frequency (f) are fixed, and j^2^ = −1 is the imaginary unit. Using the equations of Appendix B, the magnetic field ratios for vertical coplanar (VCP) and horizontal coplanar (HCP) coil orientations for a homogeneous ground are [62,63]:

(2) $${\left(\frac{H_{x}}{H_{x,p}}\right)}^{VCP}=\;\frac{-2}{\gamma^{2}s^{2}}\;\left[3-\gamma^{2}s^{2}-\left(3+\gamma s+\gamma^{2}s^{2}\right)e^{-\gamma s}\right]$$

(3) $${\left(\frac{H_{x}}{H_{x,p}}\right)}^{VCP}=\;\frac{-2}{\gamma^{2}s^{2}}\;\left[3-\gamma^{2}s^{2}-\left(3+\gamma s+\gamma^{2}s^{2}\right)e^{-\gamma s}\right]$$

Equations (2) and (3) (referred to as the full solution in the remainder of the paper) depend on the complex function γs that can be expressed in terms of the coil separation and skin depth [64], and thus in terms of the induction number:

(4) $$\gamma s=\frac{s}{\delta }\left(1+j\right)={\;N}_{b}\left(1+j\right)$$

For a horizontally n-layered subsurface, the exact Maxwell-based forward models for an EMI system in VCP and HCP orientation are written as [65,66]:

(5) $${\left(\frac{H_{x}}{H_{x,p}}\right)}^{VCP}=1-\;s^{2}\overset{\infty }{\underset{0}{\int }}R_{0}J_{1}\left(s\lambda \right)\lambda d\lambda$$

(6) $${\left(\frac{H_{z}}{H_{z,p}}\right)}^{HCP}=1-\;s^{3}\overset{\infty }{\underset{0}{\int }}R_{0}J_{0}\left(s\lambda \right)\lambda^{2}d\lambda$$

where J_0_ and J_1_ are the Bessel functions of zeroth and first order, respectively, and R_0_ is the reflection coefficient that can recursively be obtained using:

(7) $$R_{n}=\frac{\frac{\Gamma_{n}-\Gamma_{n+1}}{\Gamma_{n}+\Gamma_{n+1}}+R_{n+1}\exp\left(-2\Gamma_{n+1}h_{n+1}\right)}{1+\frac{\Gamma_{n}-\Gamma_{n+1}}{\Gamma_{n}+\Gamma_{n+1}}R_{n+1}exp\left(-2\Gamma_{n+1}h_{n+1}\right)}$$

where $\Gamma_{n}=\;\sqrt{\lambda^{2}+\gamma_{n}^{2}}$ with $\lambda^{2}$ the radial wavenumber and $\gamma_{n}^{2}$ the propagation constant of the n-th layer. The reflection coefficient R is at R_n+1_ equal to 0 [66], assuming that no fields are generated below the lowest half-space. These Maxwell-based quasi-static (no displacement currents) forward models of EMI measurements assume coils at the surface and can be adapted for elevation [65]. 

#### 2.1.2. Low Induction Number Forward Model

When H_S_ is the secondary magnetic field component measured with VCP or HCP coils, and when N_b_ << 1 (i.e., s << δ) [48], Equations (2) and (3) can be simplified to [52]:

(8) $$\left(\frac{H_{S}}{H_{P}}\right)\approx \;\frac{\;{\omega \mu }_{0}s^{2}\sigma }{4}j$$

This implies a linear relationship between H_s_/H_p_ and σ. Rearranging Equation (8) and considering that a layered subsurface is described by an apparent electrical conductivity, we can write [52]

(9) $$\sigma_{a}\;\approx \;\frac{4*Q}{{\omega \mu }_{0}s^{2}}$$

Equation (9) is often used to convert Q to σ_a_ and vice versa, and is referred to as the LIN-approximation in the following section. 

### 2.2. Exact Non-Linear EMI Conversion

Since LIN-approximation (Equation (9)) has shown limited validity (small s, low f, and σ < 12 mS/m [51,52,54]), we introduce the exact EMI conversion (EEC) to obtain accurate σ_a_ values. Just like the LIN-approximation, the EEC approach keeps the concept of an apparent electrical conductivity, i.e., a homogeneous half-space equivalent to a layered electrical conductivity distribution exists [48]. Instead of an approximation, the EEC approach uses the non-linear full solution model (Equations (2) and (3)) to calculate the response of a homogeneous half-space. When the subsurface electrical conductivity distribution is known, e.g., by a given subsurface model or due to reference DC data, Q is predicted by the Maxwell-based layered forward model (Equations (5) and (6)). To obtain the exact σ_a_ for VCP and HCP coils, the EEC approach minimizes the difference between the predicted or some measured $Q_{\mathrm{pred},\mathrm{meas}}^{\mathrm{layered}}$ of the layered subsurface and that $Q^{\mathrm{homog}}\left(\sigma_{a}\right)$ of an equivalent homogenous half-space (Equations (2) and (3)) by:

(10) $$\min\left[Q_{\mathrm{pred}.,\mathrm{meas}}^{\mathrm{layered}\;}-Q^{\mathrm{homog}}\left(\sigma_{a}\right)\right]$$

We used the non-linear numerical solver *fsolve* in Matlab (The MathWorks, Inc.) to iteratively solve for the exact σ_a_. von Hebel [25] used the EEC approach, which is similar to the approach of Guillemoteau et al. [53], who used a DualEM-21 system. The DualEM-21 uses the LIN-approximation to calculate σ_a_ from Q inside the system, such that Guillemoteau et al. [53] first back-transformed σ_a_ to Q using the LIN-approximation. Then, their Maxwell-based EMI forward models were used to estimate an exact σ_a_. The EEC approach described here also incorporates the approach of van der Kruk et al. [57], who estimated σ_a_ for large-offset EM systems with s up to 400 m using both in-phase and Q. Due to the fact that rigid-boom EMI systems with s up to 4 m measure the in-phase component less accurately [59], we have only used Q to obtain σ_a_ (and vice versa) in this study. 

### 2.3. Inversion of Multi-Coil EMI Data

We used the Maxwell-based forward models for a layered subsurface (Equations (5) and (6)) to invert the EMI data. To obtain a three-layered electrical conductivity model, we minimized the misfit using an L_1_-norm cost function [25] ΔQ for m = 1 to M coil configurations between the modeled $Q_{m}^{mod}$ and the actual $Q_{m}^{act}$. The latter is either the synthetic (simulated) or the quantitative (calibrated experimental) quadrature parts of the magnetic field ratios. 

(11) $$\Delta Q\left(\sigma_{1},\sigma_{2},\sigma_{3},h_{1},h_{2}\right)=\frac{1}{M}\overset{M}{\underset{m=1}{\sum }}\frac{\left|Q_{m}^{\mathrm{act}}-{\;Q}_{m}^{\mathrm{mod}}\left(\sigma_{1},\sigma_{2},\sigma_{3},h_{1},h_{2}\right)\right|}{\left|Q_{m}^{\mathrm{act}}\right|}$$

In particular, the EMI system used here provided m = 6 coil configurations (three VCP plus three HCP Rx). To find the global minimum, the shuffled complex evolution (SCE) inversion algorithm [67] was used to optimize the problem for unknown layer electrical conductivities (σ_1_, σ_2_, and σ_3_) and corresponding layer thicknesses h_1_ and h_2_ (the lowest zone extends to infinity). The SCE algorithm combines deterministic and probabilistic search strategies by evolving parameter combinations in periodically shuffled complexes. Parameter sets producing large misfits are discarded and newly sampled. Parameter sets producing small misfits are evolved competitively until the global minimum is found [68,69,70]. In this study, the inversion was stopped after 9000 function evaluations or when the misfit improved less than 0.01% within 10 evolution loops. The obtained inversion results were evaluated using the normalized absolute model misfit:

(12) $$\Delta \sigma =\frac{1}{N}\sum_{k=1}^{N}\frac{\left|\sigma_{k}^{ref}-\sigma_{k}^{inv}\right|}{\sigma_{k}^{ref}}\;$$

where σ^ref^ and σ^inv^ are the reference and the inverted electrical conductivities, respectively, and k = 1...N are centimeter depth increments to account for deviations in layer thickness.

**Figure 3:**
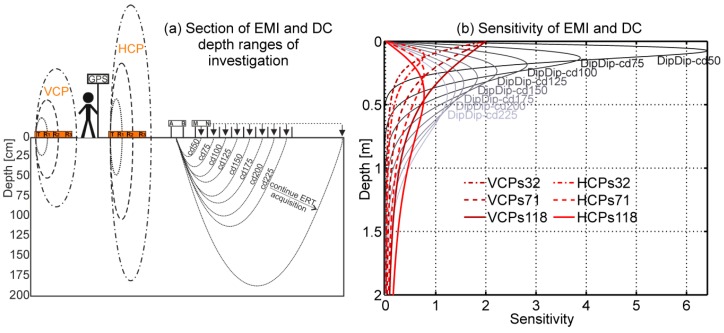
(**a**) Section of EMI and DC investigation volume respectively estimated for the EMI coil configurations and Dipole-Dipole center distances (cd) shown in (**b**). The EMI investigation volume lies above and below the surface. Note that stationary influences close to the EMI system contribute to Q and thus to σ_a_. For DC, only the subsurface is measured. (**b**) EMI and DC sensitivities; in red and dark red for the six EMI coil configurations of the CMD-MiniExplorer and in grey shadings for eight Dipole-Dipole center distances for an homogeneous half-space.

For successful inversions, a sufficiently large initial parameter search space is required. For the three-layer electrical conductivities, the range of values was from half to double of the actual σ_a_ values. For the layer thicknesses, the minimum was set to 0.15 m to ensure a stable numerical integration [24,25]. The maxima thicknesses of the two upper layers were set to 0.40 m for the first and 0.80 m for the second layer, which is based on the local sensitivity curves of rigid-boom EMI systems (see Figure 6 in [48]). The thicknesses, as set here, respect the local sensitivity of the coil configurations provided by the CMD-MiniExplorer (see Figure 3). This multi-coil EMI system carries three coplanar (VCP, HCP) receiver coils with Tx–Rx separations of s = 0.32 m, s = 0.71 m, and s = 1.18 m (referred to as s32, s71, and s118).

### 2.4. Models and EMI System Specifications for Simulation of Synthetic Data 

In the different synthetic studies, we simulated data to compare the LIN-approximation and the EEC approach. To check the conversion accuracies, we varied half-space electrical conductivities between 1 mS/m and up to 50,000 mS/m. Next, both conversion approaches we tested on layered subsurface examples with a range of electrical conductivities between 10 and 50 mS/m, which correspond to low to moderately moist sandy–silty to loamy soils [1,2,5]. In addition, the conversion approaches were studied on a saline example with σ up to 500 mS/. For these performance tests, the full solution (Equations (2) and (3)) and the Maxwell-based layered forward models of electromagnetic induction (Equations (5) and (6)) were used to simulate data for a general EMI system for VCP and HCP coils with s = 1 m, 2 m, and 4 m and f = 30 kHz. In order to invert the models for a layered electrical conductivity, data of multi-coil rigid-boom EMI system were needed. Here, we used the specifications of the CMD-MiniExplorer and studied the effect of the conversion approaches on the inversion results. This EMI system was also used to measure the experimental data, where we investigated the different conversion approaches and calibration methods. 

### 2.5. Direct Current Principle and Comparison to EMI

Direct current systems measure the potential difference between two receiver electrodes (MN) due to a current injected into the subsurface by two source electrodes (AB) to obtain σ_a_ of specific depth ranges of investigation. These depth ranges essentially increase with increasing source and receiver spacing. Among other possible electrode arrays, we used the Dipole-Dipole and Schlumberger configurations. The Dipole-Dipole array uses two pairs of neighboring AB electrodes as source and the two MN as receiver. Figure 3a displays the measurement procedure for a range increasing Dipole-Dipole center distances (cd). The MN electrodes are repeatedly moved away from AB, then AB is shifted one spacing, and so on. The Schlumberger array increases the distance between two outer source electrodes, while measuring the potential at the inner MN electrodes. This is performed along the transect line for all electrodes using the measurement management system in the case of ERT. In the case of VES, the user increases the electrodes to increase the depth range of investigation and moves then to another location. 

For multi-coil rigid-boom EMI systems, larger coil separations (s), as well as turning the system from vertical coplanar to horizontal coplanar orientation, increases the depth ranges of investigation. For VCP and HCP coils, this depth range can be approximated by 0–0.75 s and 0–1.5 s, respectively [48], with horizontal range of around 1.5 s [71]. The use of VCP and HCP coils is additionally complementary because of different local sensitivity distribution. These curves essentially describe at which depths the signal is generated. For the VCP orientation, the maximum contribution is at the surface falling to zero at depth, as displayed in Figure 3b by the dark red lines for the s32, s71, and s118. The sensitivity of the HCP coils peaks at a depth of ~0.4 s and falls to zero at the surface and at greater depth, as shown in red for the three coils. When the sensitivity cumulates to 70%, the depth range of investigation is reached. With this definition, it is obvious that the remaining 30% (below this depth range) can substantially contribute to the measured signal [12]. 

The Dipole-Dipole sensitivity curves that were obtained using the method of Barker [72] peak at the depth of 0.2 cd [49,72,73], as shown in Figure 3b. These have a similar shape as the Schlumberger curves (see Figure 5 in [73]). Thus, the use of several direct current measurements with increasing electrode distances seem appropriate to obtain similar information as EMI measurements, especially for the HCP coils and the VCP coils with larger coil separation (s71 and s118 coils).

### 2.6. Calibration of EMI Multi-Coil σ_a_ Based on Direct Current Data

Whereas the inductive EMI measurements are affected by above-surface objects that contribute to Q, and thus to σ_a_ (as indicated in Figure 3a), DC methods are affected by the subsurface only. Here, we used inverted DC data to calibrate σ_a_ measured with EMI. The vertical electrical conductivity distributions were inserted in the Maxwell-based full solution forward model (Equations (5) and (6)), together with the EMI system specifications (coil separation, coil orientation, frequency) to predict σ_a_. A linear regression between these independently obtained σ_a_ values and those measured with the EMI system provided multiplicative (m) and additive (b) calibration factors for each coil configuration (coil orientation and separation) of the multi-coil EMI system.

Since systematic errors are expected for EMI measurements, we investigated the impact of different field set-ups on the σ_a_ values measured with the EMI system. For this, σ_a_ values were recorded stepwise every 0.5 m along a 30-m-long transect using the CMD-MiniExplorer. The measurements were performed with the EMI system lying at the surface with zero-elevation. In the first set-up, the EMI system was fixed to the supplied handle (a crutch) and the operator was next to the crutch/EMI system. In the second set-up, the EMI system was mounted on a custom-made plastic sled and pulled with about 2 m distance between the operator and the sled. In this set-up, the EMI system was considered at the surface (5 mm plastic between surface and system). The GPS and data logger, as well as the cables, were always positioned in the same way. In particular, the data logger and the GPS system were fixed at positions of 0.6 m and 1 m above the multi-coil EMI system. In addition, the data cable was fixed with duct tape and straightened to remove the possible influence of a winded wire. Before the measurements, the multi-coil EMI system was allowed to warm-up for approximately 20 min, and all σ_a_ values were successively recorded within less than an hour. 

Next, we measured the ERT data using the Dipole-Dipole and Schlumberger procedure. Schlumberger was also for the vertical electrical soundings. Along a 30 m transect, ERT, VES, and EMI data were consecutively measured. The EMI measurements were performed with the CMD-MiniExplorer, first in VCP and then in HCP orientation, with a 5 Hz sampling rate. These data were resampled to 0.5 m steps. The ERT data were recorded using a Syscal Pro system (IRIS Instruments, Orléans, France) connected to 120 electrodes with 0.25 m electrode spacing. Both ERT data sets were processed with Process II (IRIS Instruments, Orléans France) using automatic- and standard deviation-based filtering. The latter filter was set to 2.5%. The ERT data sets were inverted using the robust inversion scheme of Res2Dinv (Geotomo Software, Penang, Malaysia). This scheme is suited for sharp layer boundaries [74], as expected at the test site. The VES measurements (Schlumberger array) were subsequently performed using the 4point light 10W (Lippmann Geophysikalische Messgeräte, Schaufflingen, Germany). Three VES data sets were measured with midpoints at 5, 8, and 30 m at the transect line. The separation between the current electrodes was increased from 1 to 10 m with increments of 0.5 m. These data were inverted using the approach outlined in Appendix C.

To use the electrical conductivity distribution known by ERT for σ_a_ prediction, the first and last five meters of the inverted tomogram were discarded. In these meters, the trapezoidal inversion result provides limited depth information compared to the depth range of the EMI system. For the linear regression between predicted and measured σ_a_, the obtained rectangular ERT part was re-gridded to the positions of the EMI data, with a regular spacing of 0.5 m. This resulted in 41 vertical σ profiles with layer thickness increments between 3 and 25 cm increasing from surface up to 2.12 m depth. We inserted the layered subsurface models extracted at the collocated ERT and EMI positions [42], as well as the σ distribution obtained with the VES inversion into the Maxwell-based EMI forward model (Equations (5) and (6)) to obtain Q, which was converted into σ_a_ using the EEC approach. 

### 2.7. Test Sites for EMI Calibration, Conversion, and Inversion 

We performed the measurements to develop the EMI processing chain (see Figure 1) at a field lying in the 1 km by 1 km large Selhausen agricultural CEOS LPV NASA Supersite. This Supersite lies in North-Rhine Westphalia (E: 320620 N: 5638361 Zone 32N, Germany), is part of the SFB-TR32 [75] and TERENO [76] projects, and is characterized by buried paleo-river channels. The upper terrace of the site consists of sand and gravel dominated soils with a low electrical conductivity (~5 to 15 mS/m). The lower terrace has a finer silty loam texture with an intermediate electrical conductivity (~15 to 35 mS/m) [77]. The field lies at a transition zone between the upper and lower terraces.

To test the σ_a_ calibration stability, we measured collocated ERT (Dipole-Dipole) and EMI data at various test sites and dates for a wide range of soil types and different land uses. The three-coil CMD-MiniExplorer was oriented in VCP at eighteen and in HCP at sixteen transects. At the Selhausen Supersite, nine and seven data sets were acquired at both terraces for VCP and HCP, respectively. The second test site, the Rollesbroich test site, is part of the TERENO project [76]. This is a 40 ha large grassland site in the Eifel national park (E: 309496, N: 5611678 Zone 32N, Germany) close to Belgium with Cambisols and Stagnosols with a low electrical conductivity (~2 to 10 mS/m) and depths to bedrock between 0.5 and 1.5 m [78]. Here, six data sets were measured for the VCP and HCP coils. The third site, the Scheyern test site, lies in Bavaria (E: 680100, N: 537440 Zone 32N, Southern Germany) and covers around 150 ha of arable land and grassland with loamy texture and an intermediate to high electrical conductivity (~20 to 80 mS/m) [79], where three and one data sets were acquired for the three VCP and HCP coils, respectively.

## 3. Results and Discussion 

### 3.1. Performance of the LIN-Approximation and EEC Approach on Synthetic Data 

#### 3.1.1. Comparison of the Full Solution and LIN-Approximation 

**Figure 4:**
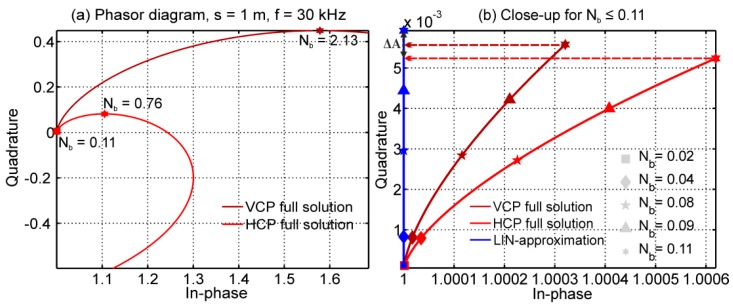
(**a**) Phasor diagram for the vertical coplanar (VCP) and horizontal coplanar (HCP) coil configuration computed using Equations (2) and (3). The coil separation was s = 1 m and the frequency f = 30 kHz. σ ranged from 0 to 50,000 mS/m. (**b**) Close-up for N_b_ ≤ 0.11 or σ ≤ 100 mS/m where the low induction number (LIN) approximation is assumed to be valid. The amplitude differences ΔA indicate that the LIN-approximation model (Equation (9)) overestimates the quadrature values even for these low values of σ.

This section investigates the differences between the full solution (Equations (2) and (3)) and the LIN-approximation (Equation (9)). We computed the magnetic field ratios for an EMI system with s = 1 m and f = 30 kHz, and the homogeneous subsurface with electrical conductivities of 1 to 50,000 mS/m. Figure 4 shows the resulting in-phase and quadrature components in a phasor diagram. A maximum is observed at N_b_ = 2.13 (σ = 38,477 mS/m) and N_b_ = 0.76 (σ = 4911 mS/m) for the VCP and HCP coil orientations, respectively. Since the LIN-approximation is valid only for N_b_ << 1, Figure 4b shows a close-up for N_b_ = 0.02 to 0.11 or σ = 1 to 100 mS/m (for s = 1 m and f = 30 kHz). The LIN-approximation consistently overestimates the quadrature values of the full solution. The overestimation is larger for HCP than for VCP coils, and generally increases with increasing electrical conductivity, increasing frequency, and increasing coil separation (see Equation (4), together with Equation (1)), which is already problematic for a wide range of soils that are typically below 100 mS/m. 

#### 3.1.2. Conversion Accuracy for Homogeneous and Layered Subsurface

**Figure 5:**
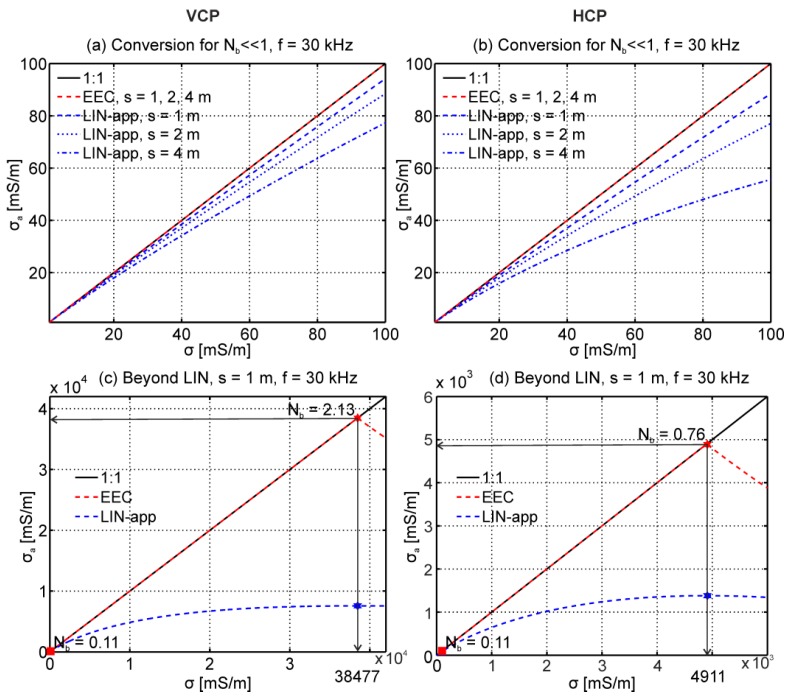
Accuracy of the low induction number-based conversion (LIN-approximation) and the exact EMI conversion (EEC) approach. The 1:1 line is provided in black. Blue lines show LIN-approximation and red lines show the EEC approach. Panels (**a**,**b**) show the conversion for VCP and HCP coils, respectively, where s = 1, 2, 4 m, f = 30 kHz, and 0 < σ < 100 mS/m. Panels (**c**,**d**) show the conversion results for VCP and HCP coils for a broader electrical conductivity range with s = 1 m and f = 30 kHz.

To illustrate the accuracy of the EEC approach, we modeled Q using Equations (5) and (6) for an EMI system with Tx–Rx separations of s = 1, 2, and 4 m in VCP and HCP orientation and f = 30 kHz. To keep N_b_ << 1 in Figure 5a,b, σ ranged between 0.1 and 100 mS/m for the VCP and HCP coils, respectively. The obtained Q was converted to σ_a_ using both the LIN-approximation and the EEC approach. The LIN-approximation already started deviating substantially at ~5 mS/m for s = 4 m. For all s, the deviation from σ_a_ = σ for the LIN-approximation increased with increasing σ. At σ = 100 mS/m, the LIN-approximation underestimated σ_a_ by around 20% for the VCP and around 40% for the HCP coils. The EEC approach (Equation (10)) performed accurately for all s and σ.

The EEC approach for larger N_b_ is shown in Figure 5c for VCP and Figure 5d for HCP coils (s = 1 m and f = 30 kHz). The EEC approach provided accurate σ_a_ values up to the maxima at 38,477 mS/m (N_b_ = 2.13) and 4911 mS/m (N_b_ = 0.76), respectively (see Figure 4). The positions of these maxima depended on the coil separation. For the largest coil separation of s = 4 m, the EEC approach provided accurate σ_a_ values up to σ = 2400 mS/m for VCP and σ = 300 mS/m for HCP coils. This is far beyond the validity of the LIN-approximation. For example, the guidelines provided with the DualEM instrument state that σ should not exceed 23 mS/m for HCP coils (s = 4 m, f = 9 kHz) to fulfill LIN conditions. 

Next, we demonstrated the accuracy of the EEC approach for a layered subsurface using three models each of three layers. Model 1 showed an increasing electrical conductivity with depth. Model 2 was reversed, with decreasing electrical conductivity with depth. Model 3 represented a saline subsurface with increasing electrical conductivity with depth. The magnetic field ratios were calculated for VCP and HCP coils (s = 1 m, f = 30 kHz) using the Maxwell-based forward model (Equations (5) and (6)). The synthetic Q values were converted into σ_a_ values using LIN-approximation and the EEC approach, as shown in Table 1. Similar to the results of Figure 5, the LIN-approximation underestimated the σ_a_ values. Also, the relative error in σ_a_ ($\mathrm{error}\left(\sigma_{a}\right)=\frac{\left|\mathrm{LIN}-\mathrm{EEC}\right|}{\mathrm{EEC}}*100)$ was larger for HCP than for VCP coils. The errors increased up to around 18% for model 3. As already shown in Figure 5, this error increases with increasing s. These results illustrate the limited range of validity of the LIN-approximation, and clearly highlight that future studies should use the EEC approach to convert between Q and σ_a_, which is valid for a broad range of induction numbers’ respective subsurface conditions (Figure 4 and Figure 5, and Table 1). 

#### 3.1.3. Impact of the Conversion Approaches on EMI Data Inversion

To test the conversion approaches in the inversion algorithm outlined above, we simulated synthetic data. The Maxwell-based EMI forward model (Equations (5) and (6)) was used to calculate Q of model 1 and 2 from Table 1. Here, it is important to note that the CMD-MiniExplorer returns σ_a_ without using the LIN-approximation, such that we convert Q to σ_a_ using the EEC approach.

Since the inversion quantity is Q (see Equation (11)), the LIN-approximation and the EEC approach back-transformed the σ_a_ values to Q. The normalized model misfit of Equation (12) was used to evaluate the obtained inversion results, as shown in Table 2. The results clearly show that the use of LIN-approximation to convert σ_a_ to Q drastically impacts the inversion outcome. 

### 3.2. Calibration Results and Impact of Calibration and Conversion on EMI Data Inversion 

#### 3.2.1. External Influences on EMI Measurements

**Figure 6:**
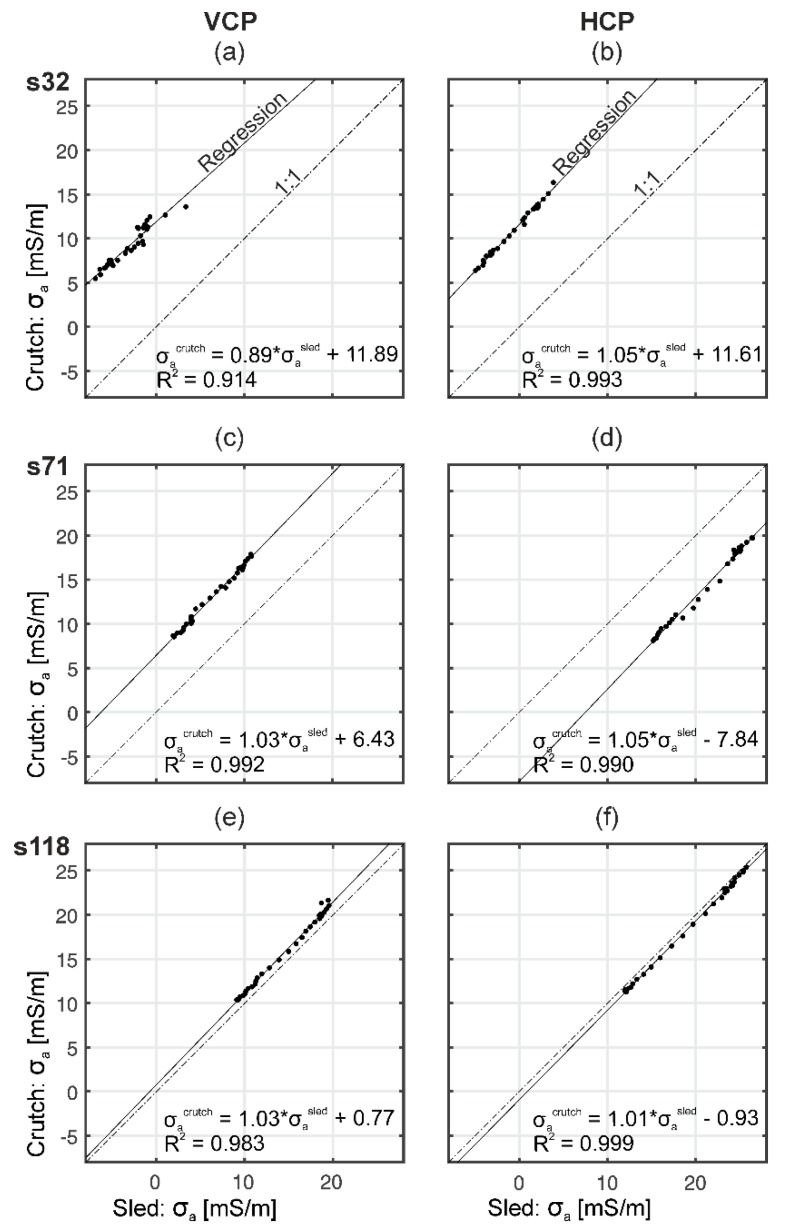
σ_a_ values measured with two different measurement set-ups using a crutch (Cr) and a sled (Sl) along a 30 m transect for VCP (**a**,**c**,**e**) and HCP (**b**,**d**,**f**) coil orientations with increasing s from top to bottom. The solid and dashed lines, respectively, show the regression and the 1:1 line.

We now move to experimental data and investigate the stationary influences affecting the EMI measurements. Here, the CMD-MiniExplorer was either attached to a crutch, which is the handle supplied by the manufacturer, or inserted in our custom-made plastic sled. Figure 6 shows the recorded values for both set-ups for the VCP and HCP coils with increasing s from top to bottom. As expected, the measurements were strongly correlated (R^2^ > 0.98, for VCPs32 R^2^ = 0.91), since they were carried out along the same transect. The slope values close to one indicate that mainly additive differences were present between the field set-ups. The σ_a_ differences were independent of the subsurface electrical conductivity and were largest for both s32 coils (around 12 mS/m). The differences decreased with increasing s, which is related to the increasing subsurface volume being sensed by the larger coil configurations. This indicates that the strength of the secondary magnetic field generated in the larger subsurface volume reduces the influence of the measurement set-up.

These results highlight that the direct surroundings of the EMI system considerably alter the signal. Therefore, we advocate the use of a standardized sled and anticipate that the use of a standardized set-up improves the reproducibility of EMI measurements. Compared to an operator-based measurement, the external influences are now more constant. However, due to the presence of stationary influences near the system, σ_a_ values recorded with EMI nevertheless require calibration.

#### 3.2.2. EMI Calibration Based on Electrical Resistivity and Vertical Electrical Sounding

**Figure 7:**
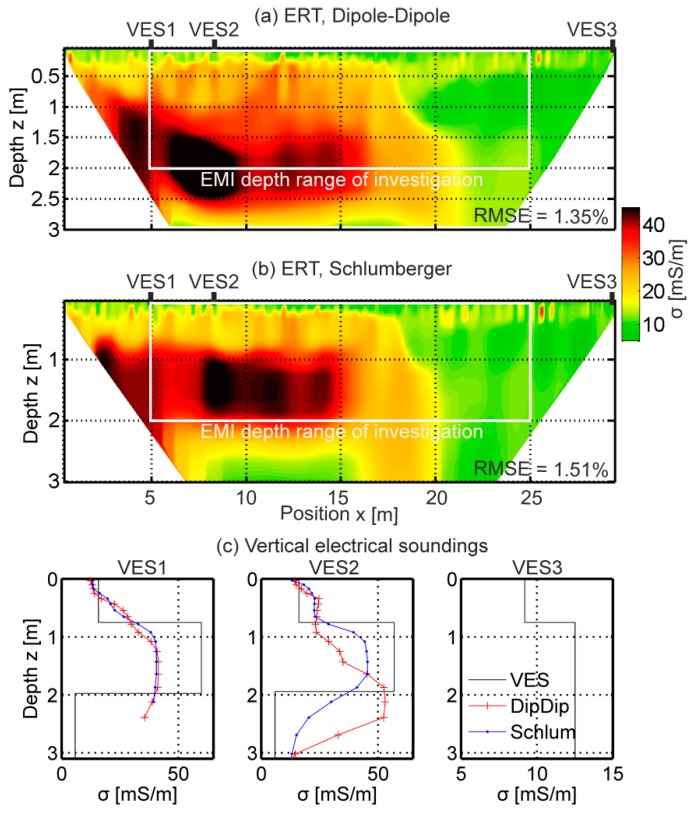
Direct current data inversion results. (**a**) ERT Dipole-Dipole inversion result. (**b**) ERT Schlumberger inversion result. (**c**) Vertical electrical sounding, VES1, VES2, and VES3, data inversion results in black. In red and blue, extracted ERT Dipole-Dipole and Schlumberger inversion results at the VES locations. At VES3, no ERT information was available. Note the scales at the VES data x-axes.

During the ERT data filtering, in total, 85 out of 6715 measurements were removed for the Dipole-Dipole and 20 out of 6120 for the Schlumberger data. The inverted tomograms with root mean squared error (RMSE) ≤1.5%, as displayed in Figure 7, showed a thin upper layer (~0.3 m) with low electrical conductivity (~10 mS/m). In the first 15 m of the transect, an approximate three-layer system with increasing electrical conductivity (up to ~45 mS/m) was present, up to around a 2.5 m and 2 m depth for the Dipole-Dipole and Schlumberger data, respectively. The inversion results for vertical electrical sounding at location x = 5 m (VES1) and at x = 8 m (VES2) at the 30-m-long transect showed similar electrical conductivity distributions as the ERT inversion results. Between 15 and 21 m, an intermediate electrically conductive (~20 mS/m) transition zone was present for the Dipole-Dipole and Schlumberger electrode arrays, although the position was slightly different in both tomograms. Between 21 and 30 m, a homogeneous zone with σ ~10 mS/m was observed. The VES inversion results at x = 30 m (VES3) showed a slight increase from σ = 9 to 13 mS/m with increasing depth. At this position, the ERT tomogram has no information. 

**Figure 8:**
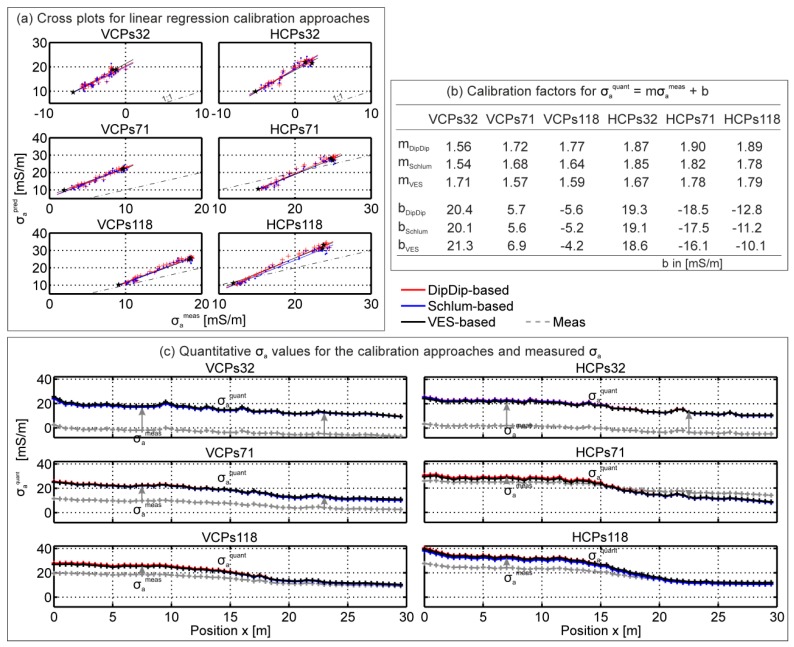
Comparison of the σ_a_ calibration approaches based on Dipole-Dipole, Schlumberger, and VES direct current methods. (**a**) Linear regression between measured and predicted σ_a_ values obtained from inverted DC data over a 30-m-long transect with Dipole-Dipole (red) and Schlumberger (blue) electrode arrays, and the inverted VES (black) data at three locations of the ERT line. (**b**) Obtained multiplicative and additive calibration factors. (**c**) Quantitative σ_a_ values based on inverted Dipole-Dipole (red), Schlumberger (blue), and VES (black) data that clearly differ from the measured σ_a_ values (grey), as indicated by the arrows.

Figure 8a shows the linear regressions between measured and predicted σ_a_ obtained from the Dipole-Dipole, Schlumberger, and VES data. The multiplicative (m) and additive (b) calibration factors obtained with the three DC methods were quite similar (see Figure 8b), even though the inverted subsurface models differed considerably. This suggests that possible errors due to non-uniqueness in the DC inversion results seem smaller compared to the calibration needed to obtain quantitative EMI data. 

The measured σ_a_ clearly deviated from the calibrated σ_a_ values, as indicated by the arrows in Figure 8c. The differences increased with increasing coil separation and were up to 20 mS/m for the s32 coils. This agrees with Figure 6, where the measurement set-up mostly influenced the small separated coils. These have small depth ranges of investigation and generate a small secondary magnetic field [80]. Since differences were present for all EMI coil configurations, calibration is necessary to correct the measured σ_a_, which can be subsequently converted using the EEC approach to obtain quantitative Q for reliable inversion. 

#### 3.2.3. Stability of σ_a_ Calibration over Different Soils

**Figure 9:**
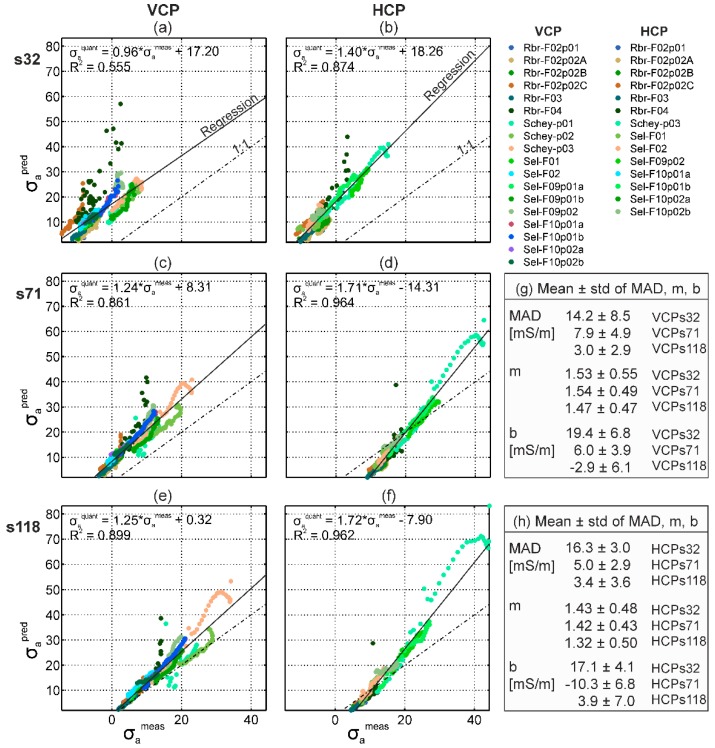
Eighteen and sixteen calibration data sets for a wide range of σ_a_ over different soils and land uses for VCP and HCP coils, respectively. (**a**–**f**) show the VCP and HCP calibration data in the left and right column, respectively, with increasing s from top to bottom. (**g**, **h**) show the mean and standard deviations of the mean absolute deviation (MAD = |( σ_a_^meas^ - σ_a_^pred^)|), of the multiplicative (m), and additive (b) calibration factors.

To test the stability of EMI data calibration based on ERT data, we acquired 18 and 16 data sets for VCP and HCP coils, respectively, at three large test sites distributed in Germany. These showed a wide range of soils. As shown in Figure 9, from the eighteen data sets acquired for the VCP coils, the linear regression between measured and predicted σ_a_ obtained R^2^ > 0.75 for fifteen VCPs32, sixteen VCPs71, and sixteen VCPs118 data sets. From the sixteen data sets of the HCP coils, R^2^ > 0.75 were obtained for thirteen HCPs32, fifteen HCPs71, and fourteen HCPs118 data sets. The calibration data sets with small R^2^ were due to a small range in σ_a_ in the selected 30 m calibration line, which is problematic for calibration using linear regression. Here, a shift factor (mean difference between σ_a_^meas^ and σ_a_^pred^) may be used for calibration. 

Overall, Figure 9 shows that data from the different sites, soils, and dates approximately line up from relatively low to relatively high σ_a_ values with an overall R^2^ > 0.85, except for VCPs32, with a lower R^2^ of 0.56. The VCPs32 is most sensitive to the upper soil centimeters where comparably large heterogeneity is present (voids, air, biopores, etc.), and can only generate weak secondary magnetic fields. Also, the use of finite length electrodes in DC data acquisition reduces the measured potential difference compared to the assumed point electrodes in the data inversion. This may have systematically biased the σ_a_ values measured by the DC methods for shorter electrode spacing [81]. For future calibration of coils with small separation, the DC data should contain more information about the upper subsurface centimeters. This can be achieved using shorter electrode spacing, thin point-like electrodes, or modeling of finite-length electrodes in the inversion.

Figure 9 also shows the mean and standard deviation of the mean absolute difference (MAD) between measured and predicted σ_a_ values. In addition, the mean and standard deviation of the m and b calibration factors are shown for each EMI coil configuration. Consistent with the findings in Figure 6, Figure 8b and Figure 9, the b factors and the MAD were largest for the smallest coil separation and decreased with increasing s. The mean m was around 1.5 ± 0.5 for all coil configurations over a reasonable range of soils, indicating the stability of the approach. Variations in the m values at individual sites may be related to the relatively narrow σ_a_ range (point clouds) used to estimate this factor. In addition, it is possible that the m factor was affected by the electrical conductivity distribution with depth.

### 3.3. Impact of Calibration and Conversion on EMI Data Inversion

**Figure 10:**
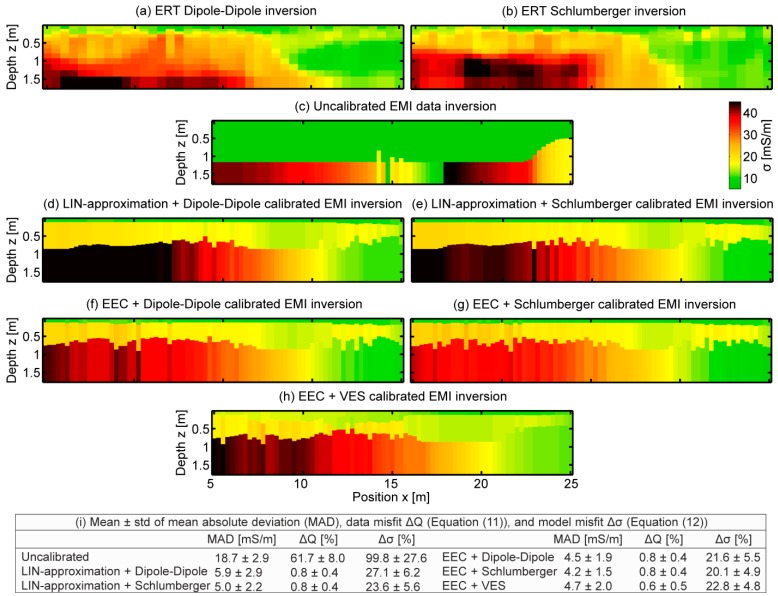
Comparison of calibration and conversion approaches in multi-coil EMI data inversions. (**a**,**b**) show the reference ERT tomograms using Dipole-Dipole and Schlumberger electrode arrays, respectively. (**c**) shows the EMI inversion of uncalibrated data. (**d**,**e**) show the EMI inversion of Dipole-Dipole and Schlumberger-based calibration, together with the LIN-approximation. (**f**,**g**) show the corresponding EMI inversion results using the EEC approach. (**h**) shows the VES-based calibrated results converted with EEC. (**i**) shows mean and standard deviation of the mean absolute deviation (MAD), as well as of the data misfit ΔQ (Equation (11)), and the model misfit Δσ (Equation (12)).

To study the impact of the conversion and calibration on the inversion of experimental EMI data, we calibrated the σ_a_ values measured with the CMD-MiniExplorer using the three calibration methods, and used the LIN-approximation and the EEC approach, as outlined in Figure 1, to convert the quantitative σ_a_ values to Q prior to inversion. In Figure 10a,b, the Dipole-Dipole and Schlumberger tomogram cuts of Figure 7, matching the investigated depth ranges of the EMI system, are presented as reference for the intrinsic electrical conductivity distributions. Clearly, the inversion of the uncalibrated EMI data shown in Figure 10c deviated strongly from these reference data. 

Next, we compared the performance of the LIN-approximation and the EEC approach in the inversion of the EMI data obtained with the CMD-MiniExplorer. Figure 10d,e, respectively, shows the inversion results for the LIN-approximation based on the Dipole-Dipole and Schlumberger calibration. Compared to the results using the EEC approach, together with the Dipole-Dipole (Figure 10f) and Schlumberger-based calibration (Figure 10g), as well as with the VES-based calibration (Figure 10h), the lowest zone obtained much higher electrical conductivities. These were also larger than the ERT references. Already, the synthetic data inversion using the LIN-approximation in model 2 (increasing electrical conductivity with depth) presented in Table 2 showed too high inverted σ values in the lowest zone compared to the reference model. Since the LIN-approximation overestimates the quadrature amplitude, as shown in Figure 3, this leads to erroneous data. This error will increase with increasing electrical conductivity, as observed in the saline example shown in Table 1. 

To quantify the performance of the different calibration and conversion methods, Figure 10i shows the MAD between the ERT references and the EMI inversion results. Further, the mean of the data misfit ΔQ, Equation (11), and the mean model misfit Δσ of Equation (12), are shown. As expected, the uncalibrated EMI data inversion resulted in the largest differences and misfits. The MAD, ΔQ, and Δσ were smaller for the EEC than for the LIN-approximation. Since the σ values were relatively low at this test site and the errors due to conversion increase with increasing electrical conductivity as shown in the synthetic studies, we expect larger errors for test sites with soils of larger σ.

## 4. Conclusions

We presented a novel data processing workflow that consists of conversion, calibration, and inversion of EMI data for reliable and high resolution large-scale subsurface imaging and characterization. For an accurate conversion of the quadrature part of the secondary magnetic field (Q) to the apparent electrical conductivity (σ_a_), we proposed an exact EMI conversion. This EEC approach uses the information of the full solution for a homogeneous subsurface to obtain σ_a_, instead of a truncated Taylor expansion as used in the (very) low induction number-based conversion (LIN-approximation). The LIN-approximation already provided erroneous results when σ ≥ 5 mS/m for s = 1 m and f = 30 kHz. For this s and f, the EEC approach performed accurately up to ~40,000 mS/m and ~5000 mS/m for VCP and HCP coils, respectively. We consequently emphasize that Q should be converted to σ_a_ using the EEC approach to obtain the most accurate σ_a_ estimates. 

EMI measurements require calibration to account for the effect of measurement influences, such as the field set-up surrounding the system. The ERT-based (Dipole-Dipole and Schlumberger) and VES-based calibrations resulted in similar (multiplicative, m, and additive, b) calibration factors for a wide range of different soils. In addition, the differences between uncalibrated/measured and calibrated σ_a_ values are substantial, whereas possible biases due to DC data inversions play a minor role. For a successful EMI data calibration, the DC and EMI data need to cover the entire σ_a_ range of a test site. This range should be >3 mS/m to avoid point clouds in the linear regression, and the σ_a_ should be >5 mS/m to generate sufficiently large signals for the EMI system. When acquiring calibration data using an ERT transect, the electrical conductivity distribution should vary both horizontally and vertically along that line. For VES measurements, relatively homogeneous profiles of low to high σ_a_ across the test site should be selected. This consequently reduces ambiguity in the inversions. Here, the two-dimensional (2D) ERT inversion may still be less ambiguous as the 1D VES inversion result with the cost of more exhaustive data acquisition and less flexibility in the field.

The m and b calibration factors allow the following interpretations. The m factors were around 1.5, highlighting the consistency of the approach. The b factors were largest for the smallest depth ranges of investigation, indicating that the stationary influences mostly impact the coils with small separation. These coils can deliver important information of the upper subsurface, e.g., for remote sensing applications. To fully understand the interaction between soil heterogeneity in the upper few centimeters and the EMI and DC signal, further research may use point-like electrodes and/or model finite-length electrodes in the DC inversion. 

Summarizing, all calibration results show that DC information is suitable to calibrate the σ_a_ values measured with EMI. The inversion of calibrated and EEC converted EMI data were closest to the reference images. The novel and complete processing chain introduced here performs calibration, accurate conversion, and quantitative inversion of the intrinsic electrical conductivity of certain layers. The different strategies of the processing workflow proposed for the EMI system considered here can be used to build different processing combinations for other types of EMI systems, such that high resolution large-scale (quasi-)3D studies can be performed with relatively low effort. In that way, quantitative joint interpretations with data of other geophysical sensors are possible, as well as combinations of the depth-specific properties with, e.g., data of remote sensing platforms.

## Figures and Tables

**Table 1:** Converted σ_a_ values obtained using LIN-approximation and EEC approach for three-layered models with increasing and decreasing σ_i_, i = 1,2,3, with depth. h_1_ and h_2_ are the thicknesses of the first and second layer, respectively. The Q were simulated for an EMI system with s = 1 m and f = 30 kHz using Equations (5) and (6). The error is $\frac{\left|\mathrm{LIN}-\mathrm{EEC}\right|}{\mathrm{EEC}}\;\times \;100$.

Model	σ_1,_ σ_2,_ σ_3_ [mS/m]	h_1_, h_2_ [m]	LIN-VCP [mS/m]	EEC-VCP [mS/m]	Error LIN-VCP [%]	LIN- HCP [mS/m]	EEC-HCP [mS/m]	Error LIN-HCP [%]
**1**	10, 20, 50	0.3, 0.5	22.3	23.0	2.8	32.8	30.6	6.6
**2**	50, 20, 10	0.3, 0.5	29.9	30.9	3.2	19.6	18.6	5.1
**3**	20, 100, 500	0.3, 0.5	128.3	137.7	6.8	240.7	197.7	17.9

**Table 2:** EMI data inversion results for two three-layer models using LIN-approximation and the EEC approach to convert data.

Parameter/Approach	σ_1_ [mS/m]	σ_2_ [mS/m]	σ_3_ [mS/m]	h_1_ [m]	h_2_ [m]	Δσ [%]
**Model 1**	10.0	20.0	50.0	0.3	0.5	
**LIN**	9.8	13.5	61.4	0.2	0.7	25.0
**EEC**	10.0	19.8	49.9	0.3	0.5	1.4
**Model 2**	50.0	20.0	10.0	0.3	0.5	
**LIN**	50.2	21.6	12.0	0.3	0.4	14.6
**EEC**	50.0	22.0	10.6	0.3	0.4	9.6

## Appendix A. EMI and DC Similarities

A critical concern when using direct current methods for the σ_a_ calibration may be that DC methods are galvanically coupled to the subsurface and use alternating currents of a few Hertz (Hz), while EMI is inductively coupled and uses alternating currents in the kHz range. In DC methods, the current (I) flows due to a potential difference (U) through the earth with a resistance (R) or conductance (G), such that I $=\;\frac{U}{R}=U\cdot G$, assuming an isotropic homogeneous layer. For EMI, the electromagnetic force (emf) induces I in each layer, such that I∝ emf·G [48] and all current flow is horizontal [48]. The frequency is sufficiently low, such that self-inductance of, and mutual inductance between, layers are assumed negligible [48] and the currents flow deep in/through the conductor [82]. Therefore, when the subsurface layers are reasonably electrically conductive (no resistive layers), the conductance determines the current flow for both DC and EMI.

## Appendix B. EMI Primary and Resultant Magnetic Field of Homogeneous Ground

The EMI systems use a transmitter coil to generate a primary magnetic field, where vertical and horizontal transmitter coils act as horizontal and vertical magnetic dipoles, respectively. VCP coils produce a horizontal magnetic dipole. HCP coils produce a vertical magnetic dipole. The x and z components of the secondary field over a homogeneous half-space are expressed as [66]:

(A1) $$H_{x}{}^{\mathrm{VCP}}=\;\frac{m}{2{\pi \gamma }^{2}s^{5}}\;\left[3-\gamma^{2}s^{2}-\left(3+\gamma s+\gamma^{2}s^{2}\right)e^{-\gamma s}\right]\;,$$

(A2) $$H_{z}{}^{\mathrm{HCP}}=\;\frac{-m}{2{\pi \gamma }^{2}s^{5}}\;\left[9-\left(9+9\gamma s+4\gamma^{2}s^{2}+\gamma^{3}s^{3}\right)e^{-\gamma s}\right]\;,$$

where m is the magnetic dipole moment, γ is the propagation constant, and s is the coil separation. The primary magnetic field component is [82]:

(A3) $$H_{P}^{VCP,HCP}=\;\frac{-m}{4\pi s^{3}}\;.$$

In this paper, the ratio of the secondary and primary magnetic field component for a homogeneous ground is termed as the full solution model.

## Appendix C. Direct Current Data Modeling and Inversion

Direct current methods such as electrical resistivity tomography (ERT) or vertical electrical sounding (VES) use two electrodes to generate a current flow and two potential electrodes to measure the apparent resistivity (ρ_a_), i.e., the reciprocal of σ_a_. ERT uses multiple electrodes along a line connected with a multi-core cable to a control unit to measure the lateral and vertical ρ_a_ distribution. VES increases the electrode spacing at a specific location to obtain the vertical ρ_a_ distribution [60]. Using inversion techniques for ρ_a_ data, the layered electrical conductivity distribution can be obtained. For the inversion of VES data, we implemented an inverse-modeling scheme in Matlab. According to Koefoed [83], the ρ_a_ for a number of homogeneous and isotropic layers can be obtained using:

(A4) $$\rho_{a}=\;{\left(\mathrm{AB}/2\right)}^{2}\int_{0}^{\inf}T\left(\lambda \right)J_{1}\left(\lambda s\right)\lambda \;d\lambda \;,$$

where

(A5) $$T_{n}\left(\lambda \right)=\;\frac{T_{i+1}\left(\lambda \right)+\rho_{i}\tan h\left(\lambda h_{i}\right)}{1+\;T_{i+1}\left(\lambda \right)\frac{\tanh\left(\lambda h_{i}\right)}{\rho_{i}}}\;,\;i\;=\;n-1,\;\ldots ,\;1.$$

Here, AB/2 is half of the outer electrode spacing, J_1_ is the Bessel function, and λ the integration variable. The resistivity transform function T(λ) is obtained recursively, where n denotes the number of layers. The ρ_i_ and h_i_ are the i-th electrical resistivity and the i-th layer thickness, respectively [84,85]. To find the global minimum in the inversion, the SCE algorithm was used. The inversion of ERT data was performed using the robust inversion scheme of Res2DInv software (Geotomo, Malaysia), such that we either obtain the electrical conductivity distribution along a transect for ERT or at a certain location for VES, which is used in the Maxwell-based forward model of electromagnetic induction together with the EMI system specification to predict σ_a_ values.
